# Interplay between Inflammation and Pathological Bone Resorption: Insights into Recent Mechanisms and Pathways in Related Diseases for Future Perspectives

**DOI:** 10.3390/ijms23031786

**Published:** 2022-02-04

**Authors:** M Alaa Terkawi, Gen Matsumae, Tomohiro Shimizu, Daisuke Takahashi, Ken Kadoya, Norimasa Iwasaki

**Affiliations:** Department of Orthopedic Surgery, Faculty of Medicine and Graduate School of Medicine, Hokkaido University, Kita-15, Nish-7, Kita-ku, Sapporo 060-8638, Japan; gen_matsu_mae@yahoo.co.jp (G.M.); simitom@wg8.so-net.ne.jp (T.S.); rainbow-quest@pop02.odn.ne.jp (D.T.); kadoya@rf7.so-net.ne.jp (K.K.); niwasaki@med.hokudai.ac.jp (N.I.)

**Keywords:** bone, inflammation, remodeling, pathological bone resorption, bone osteolytic diseases

## Abstract

Bone is a mineralized and elastic connective tissue that provides fundamental functions in the human body, including mechanical support to the muscles and joints, protection of vital organs and storage of minerals. Bone is a metabolically active organ that undergoes continuous remodeling processes to maintain its architecture, shape, and function throughout life. One of the most important medical discoveries of recent decades has been that the immune system is involved in bone remodeling. Indeed, chronic inflammation has been recognized as the most significant factor influencing bone homeostasis, causing a shift in the bone remodeling process toward pathological bone resorption. Bone osteolytic diseases typified by excessive bone resorption account for one of the greatest causes of disability worldwide, with significant economic and public health burdens. From this perspective, we discuss the recent findings and discoveries highlighting the cellular and molecular mechanisms that regulate this process in the bone microenvironment, in addition to the current therapeutic strategies for the treatment of osteolytic bone diseases.

## 1. Introduction

Bone is a highly vascu larized and mineralized tissue that naturally maintains its mass, microarchitecture, shape, and function through an orchestrated process called bone remodeling. This process requires tight coordination between four types of cells, all of which reside in the bone. These include osteoblasts and osteocytes of mesenchymal origin, and osteoclasts and osteal macrophages (Osteomacs) of hematopoietic origin that interact with each other via endocrine, paracrine, and autocrine signaling systems [1,2]. Importantly, disruption of the bone remodeling process frequently occurs during inflammation, which plays a major role in skewing this balanced process toward bone resorption through promoting osteoclast activity [3]. The involvement of immune pathways in bone pathophysiology has been recognized since the early 1970s, when researchers discovered that inflammatory cytokines are able to promote osteoclastogenesis, leading to pathological bone resorption. These findings resulted in the emergence of a new important research field called osteoimmunology, which further provides a molecular basis for the discovery of therapeutic agents that are currently used in clinics for the treatment of bone disorders [4,5,6].

Pathological bone resorption has been documented in at least 100 chronic inflammatory conditions, including rheumatoid arthritis, spondylarthritis, periodontitis, carcinoma metastasis, aseptic loosening of joint arthroplasty, Crohn’s disease, systemic lupus erythematosus, inflammatory bowel disease, celiac disease, and cystic fibrosis [5,7,8,9,10]. Many factors that are classically considered as immune-related molecules have been found to be crucial in bone remodeling. As such, interleukins 1 beta (IL-1β), 6 (IL-6) and 17 (IL-17), and tumor necrosis factor alpha (TNF-α) are the main stimulators of osteoclastogenesis. It is known that these cytokines are involved in and promote the expression of key factors related to osteoclast differentiation, namely the receptor-activator of nuclear factor kappa B ligand (RANKL) and macrophage colony stimulating factors (M-CSF), leading to pathological bone resorption [11,12,13,14,15]. It is also known that chemokines, another group of inflammatory mediators that are known to play a role in enhancing cellular migration, tissue remodeling, and angiogenesis, have a substantial impact on osteoclastogenesis. Several lines of evidence suggest that chemokines such as C-C motif chemokine ligands (CCLs): CCL3, CCL5, CCL9, CCL19, CCL20, CCL21, and the C-X3-C motif chemokine ligand (CX3CL1) and the X-C motif ligand 1 (XCL1) potentiate the differentiation of osteoclasts though the activation transcription factors and positive regulators of osteoclastogenesis [16,17,18,19,20]. In addition to the effects of these inflammatory mediators on osteoclast activity, they also affect other cell types that reside in the bone microenvironment; as such, a number of these inflammatory mediators have been shown to inhibit osteoblast differentiation and induce osteocyte apoptosis, resulting in a reduction in bone formation, mineralization, and density [21,22,23,24,25,26,27,28]. 

On the other hand, immune cells produce factors that can inhibit osteoclast differentiation through blocking RANKL signaling and other positive regulators of osteoclastogenesis. Of these factors, osteoprotegerin (OPG), a soluble decoy receptor for RANKL, is expressed by immune and bone cells and is generally considered to be a master regulator of osteoclastogenesis, since it binds RANK and blocks RANKL signaling and osteoclast differentiation [29,30]. Therefore, the OPG/RANKL/RANK axis has received increasing interest as a therapeutic target for a broad range of bone loss-related diseases. In fact, the importance of this axis has been demonstrated in transgenic mice based on the finding that RANK- or RANKL-deficient mice exhibit severe osteopetrosis and OPG-deficient mice develop a severe form of osteoporosis typified by the loss of trabecular and cortical bone [29,30]. Other immune factors including IL-4, IL-10, IL-27, interferons (IFNs), annexin A1 (AnxA1), and cardiotrophin-like cytokine factor 1 (CLCF1) act as negative regulators of osteoclastogenesis and act by blocking the activation of transcription factors needed for cell differentiation [31,32,33,34,35,36]. These extremely important findings highlight the importance of inflammatory pathways as critical targets in both the prevention and therapy of bone osteolytic diseases.

## 2. Cellular and Signaling Networks Linking Inflammation and Pathological Bone Resorption 

**Figure 1:**
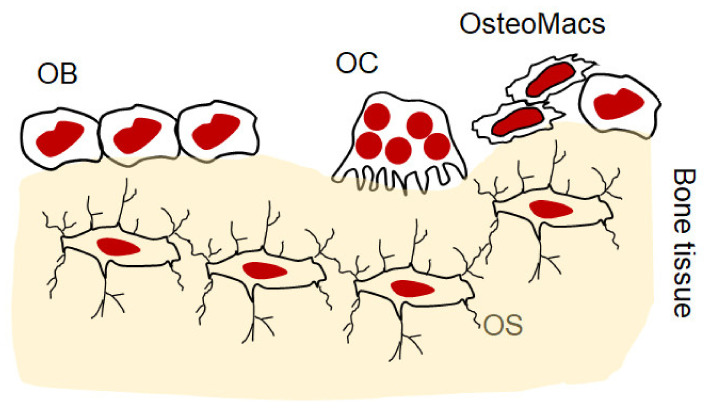
Schematic diagram illustrating the cellular entities in bone microenvironment. OB: osteoblast, OC: osteoclast, OS: osteocytes, osteomacs: osteal macrophage.

The bone microenvironment is composed of cellular entities, including osteoblasts and osteocytes of mesenchymal origin, and osteoclasts and osteomacs of hematopoietic origin (Figure 1). The interplay between these cells is tightly regulated, and maintaining the remodeling process and homeostatic condition in the bone is dependent on a complex set of paracrine and endocrine signals. Disruption of this regulation impairs the physiological function of these cells, becoming a pathological process typified by increasing bone loss. Inflammation is a major factor in impairing this physiological process of bone remodeling and, in a variety of bone osteolytic diseases, skews it toward bone resorption [26].

### 2.1. Osteoclastogenesis and Physiological and Pathological Functions of Osteoclasts

Osteoclasts are multinucleated cells derived from mononuclear precursors of monocytes and macrophages that fuse and form specialized bone-resorbing cells that are located on the endosteal bone surface and the periosteal surface beneath the periosteum. They are the key participants in bone remodeling, and their physiological activity is crucial for maintaining skeletal structure and function by removing damaged and old bone. Indeed, a decline in the number of mature osteoclasts leads to a condition called osteopetrosis, which is characterized by irregular bone formation with high density that is prone to fracture. The duration of the resorption phase is limited and characterized by recruiting osteoclast precursors to the remodeling site, followed by the reversal phase that is characterized by the disappearance of osteoclasts that are replaced by osteoblasts for bone formation [1,3]. Nonetheless, the hyperactivity of osteoclasts causes excessive bone loss and disrupts the bone architecture, eventually leading to bone fragility, which is the principal feature of a broad range of diseases, including osteoporosis, rheumatoid arthritis, periodontitis, and aseptic loosening [8,9].

**Figure 2:**
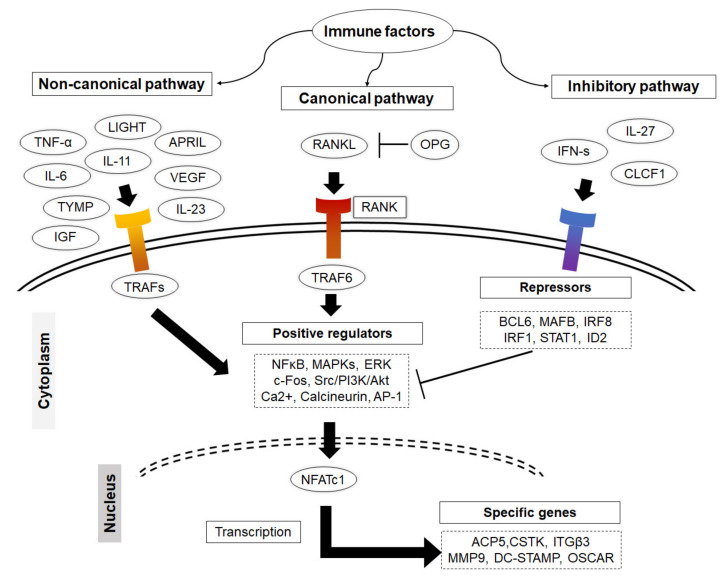
Illustration of how immune factors impact on osteoclastogenesis. Immune cells produce a variety of factors that promote osteoclastogenesis via both canonical and non-canonical pathways. They activate TRAFs that initiate the action of NFκB and the c-src /PI 3-kinase/Akt signal transduction pathways. These lead to the activation of downstream molecules c-Fos, Fos B, FRA-1, FRA-2 and c-Jun, JunB, JunD, followed by the induction of the master regulator of osteoclast differentiation NFATc1. Some of the other immune factors are able to initiate the autoregulation system for RANKL signaling by inducing the expression of transcriptional repressors to restrain cell differentiation.

The canonical pathway of osteoclast formation is dependent on RANKL, which is mainly expressed by osteoblasts, stromal cells, synoviocytes and lymphocytes to induce cell fusion and differentiation [37]. Nonetheless, increased RANKL expression in bone mainly occurs as a result of a local increase in the levels of osteotropic hormones, including 1,25-dihydroxy vitamin D3 (1,25(OH)2D3), parathyroid hormone (PTH), and pro-inflammatory cytokines such as TNF-α, IL-1β and IL-6. Following the cleavage of RANKL by certain metalloproteinases, the soluble form (sRANKL) binds its receptor, RANK, on the surface of osteoclast progenitors that recruit the adaptor protein TNF-related factor 6 (TRAF6). The activation of TRAF6 initiates the action of a range of signal transduction pathways including the NFκB and the c-src /PI 3-kinase/Akt pathways. These events lead to the activation of downstream molecules; Fos (c-Fos, Fos B, FRA-1, FRA-2) and Jun (c-Jun, JunB, JunD), followed by the induction of the nuclear factor of activated T-cells cytoplasmic 1 (NFATc1) [3,38,39,40,41,42]. NFATc1, the master regulator of osteoclasts, mediates fusion, maturation, activation, and function (Figure 2). M-CSF, which is expressed in fibroblasts, osteoblasts, and activated macrophages is another essential growth factor that plays an essential role in the survival and maturation of osteoclasts via activating the expression of RANK on the myeloid cells. A mutation in M-CSF causes developmental bone defects in mouse bone and a reduction in the number of mature osteoclasts as well as tissue macrophages [43,44]. It should also be noted that the differentiation of osteoclasts requires tight coordination between the RANKL pathway and costimulatory signals, namely Ca2+ signaling. RANK signaling is associated with the activation of immunoreceptor tyrosine-based activation motif (ITAM) signaling where the Fc receptor common γ subunit (FcRγ) and the DNAX-activating protein-12 (DAP12) act as adapter proteins on the surface of osteoclast precursors, thus facilitating intracellular signaling. ITAM activation promotes the mobilization of intracellular Ca2+, which that facilitates the nuclear translocation and auto-amplification of NFATc1 [45]. In fact, the induction of NFATc1 results in the upregulation of a number of essential genes, including β3 integrin and c-Src, that mediate the adhesion of osteoclasts to the bone surface, and ATP6i, acid phosphatase 5 (ACP5), DC-STAMP, cathepsin K (CTSK), matrix metalloproteinase 9 (MMP9), and latent-transforming growth factor beta-binding protein 3 (LTBP3), that facilitate osteoclast function [46,47]. 

Although RANKL signaling is the major pathway of osteoclastogenesis, there is a growing body of evidence to suggest that the non-canonical pathway is involved in this process, since certain growth factors substitute RANKL and induce cell fusion and differentiation [37]. However, the significance of the non-canonical pathway in osteoclast differentiation remains to be determined, because current data are mainly derived from in vitro studies. Among these growth factors, TNF-α, which is expressed by macrophages, monocytes and T cells, is known to promote osteoclastogenesis in a dose-dependent manner [48,49]. The tumor necrosis factor superfamily, including TNFSF14, also known as LIGHT (lymphotoxin exhibiting inducible expression and competing with herpes simplex virus glycoprotein D for herpesvirus entry mediator, a receptor expressed by T lymphocytes), APRIL (a proliferation inducing ligand), TNFSF13, and BAFF (B cell activating factor), have been documented as osteoclastogenic factors that are capable of inducing osteoclast production in cultures of monocyte precursors [50,51,52,53]. Other immune factors that exhibit the ability to induce osteoclastogenesis in monocyte cultures include transforming growth factor β (TGF-β), insulin-like growth factor (IGF), thymidine phosphorylase (TYMP), vascular endothelial growth factor (VEGF), IL-6, IL-11, and IL-23 [52,54,55,56,57,58,59,60]. It appears that these factors promote osteoclast differentiation through turning on adaptor proteins rather than TRAF6, activating other positive regulators of osteoclastogenesis, leading to cell fusion and maturation [50,53]. Although osteoclastogenic humoral factors are not as potent as RANKL in inducing osteoclast formation, in terms of the size of the formed cell and their resorptive activity, their potential role as osteoclastogenic factors should not be neglected, especially because they are highly expressed around osteolytic bone and joint tissues by immune cells in inflammatory diseases [50,52,57,59,61,62]. Taken together, inflammation acts as a switch that turns on pathological bone resorption through promoting osteoclastogenesis via both canonical and non-canonical pathways. 

On the other hand, osteoclasts initiate the autoregulation system for RANKL signaling by inducing the expression of transcriptional repressors to oppose the positive regulators of osteoclastogenesis and restrain cell differentiation. This process is tightly regulated and appears to be physiologically critical for the maintenance of normal bone mass and osteoclast activity. Importantly, mature osteoclasts produce IFN-β, which acts as a negative-feedback regulator that suppresses osteoclast differentiation by interfering with the RANKL-induced expression of c-Fos [63]. The importance of IFN signaling in negative feedback signaling and homeostasis was confirmed in mice that are deficient in each of IFN-signaling pathway components, including IFN receptor 1 (IFNAR1), interferon regulatory factors (IRF1 and IRF9), and STAT1, resulting in a decreased bone density and increased numbers of osteoclasts in bone tissue compared to wild-type mice [34]. Therapeutically, the administration of recombinant IFNs including (IFN-β or IFN-γ) inhibits bone loss and increases bone density in patients with osteolytic bone diseases, including rheumatoid arthritis and osteoporosis [34,63,64,65,66]. Likewise, the activation of IFN signaling by administration IL-27 and CLCF1 inhibits osteoclast differentiation and protects against bone loss in experimental mouse models for inflammatory bone loss and osteoporosis [35,36,67,68,69,70]. Other transcriptional repressors include v-Maf musculoaponeurotic fibrosarcoma oncogene homology B (MAFB), interferon regulatory factor 8 (IRF-8), B lymphocyte–induced maturation protein 1 (BLIMP1), and B cell lymphoma 6 (BCL6) [71]. Their important function in maintaining bone mass has been documented in deficient mice, including IRF-8 deficient mice, and mice doubly mutant in Blimp1 and Bcl6 exhibit decreased bone mass with increased osteoclastogenesis [72,73,74]. In an analogous manner, immune cells, upon stimulation, express certain immune factors that are able to inhibit osteoclastogenesis though activating osteoclast transcriptional repressors [71]. 

In addition to the resorptive activity of osteoclasts, they also produce a number of factors including BMP6, CTHRC1, EFNB2, S1P, WNT10B, SEMA4D, and cardiotrophin-1 (CT-1) that impact nearby cells, namely osteoblasts and osteocytes in terms of differentiation and function [75]. In support of these findings, the osteoclast-specific deletion of Cthrc1 results in reduced bone formation and impaired bone remodeling [76,77]. CT-1, another molecule that is secreted by osteoclasts, belongs to the interleukin-6 (IL-6) family and is known to promote bone formation through the activation of the LIF receptor (LIFR) and GP130 signaling, based on the observation that mice deficient in Ct-1, Lifr and Gp130 exhibit a low quality of bone with a reduced number of bone surface osteoblasts [78,79,80]. Moreover, extracellular vesicles derived from osteoclasts exert anabolic function in the bone microenvironment by facilitating cell–cell communication and promoting osteoblastic bone formation [81]. These collective findings highlight the distinct role of osteoclasts in maintaining bone homeostasis, as they participate in physiological and pathological bone resorption, regulate the function of osteocytes and osteoblasts and promote bone formation. However, inflammation promotes osteoclastogenesis via the activation the canonical and the non-canonical pathways and disrupts the regulatory function of osteoclasts on osteocytes and osteoblasts, thus leading to the development of bone loss and osteolytic diseases.

### 2.2. Inflammation and the Regulatory Functions of Osteoblasts and Osteocytes

Osteoblasts are highly specific cells that produce a collagenous matrix and mediate the maturation and mineralization of the bone matrix through the production of type 1 collagen, the most abundant matrix protein, alkaline phosphatase, and other non-collagenous proteins, resulting in the formation of new bones. They also function to regulate the levels of calcium and phosphate ions in bone. Osteoblasts comprise 5% of all bone cells and are usually found on the surface of the bone as bone lining cells or embedded within their own matrix, where they further differentiate into interconnected osteocytes. Osteoblasts are derived from bone marrow mesenchymal stem cells, and their maturation is mainly mediated by the WNT pathway. The release of TGF-β from osteoblasts exerts a negative feedback action on bone resorption through upregulating OPG and suppressing the RANKL-signaling pathway in vitro. Importantly, the Runt-related transcription factor 2 (RUNX2) and osterix (OSX; known as SP7) are critical for osteoblast differentiation and differentiated osteoblasts express unique markers including collagen type I alpha 1 chain (ColIA1), alkaline phosphatase (ALP), bone sialoprotein (BSP), and osteocalcin (OCN) [75,82].

Osteocytes, a subset of osteoblasts that are embedded within the mineralized bone matrix, account for 95% of all cells in mature bone tissue and play an essential role in calcium phosphate homeostasis through the vitamin D signaling pathway. Osteocytes form communication networks through dendritic processes forming lacunar–canalicular networks that connect osteocytes with each other and with bone surface cells as well as marrow. This network system allows the cells to receive nutrition and to sense any changes within the microenvironment of bone and to coordinate the function of osteoblasts and osteoclasts in response to environmental changes. The mechanosensory function of osteocytes underlines their essential role in the adaptation of bone to environmental changes such as mechanical forces [83,84]. Moreover, osteocytes are the main regulators of the canonical WNT/β-catenin signaling pathway, which is critical for the bone remodeling process [85,86,87,88,89].

Osteoblasts and osteocytes produce a variety of regulatory molecules mediating the balance between bone formation and bone resorption. Specifically, osteoblasts produce osteoclastogenic factors including RANKL, M-CSF, vascular endothelial growth factor A (VEGFA), and Wnt family member 5A (WNT5A), and anti-osteoclastogenic factors including OPG, semaphorin 3A (SEMA3A), and WNT16. The importance of these factors has been documented in transgenic mice, where Sema3a-deficient mice were reported to exhibit a severe osteopenic phenotype in trabecular and cortical bones with an increase in osteoclast number [90]. Likewise, the conditional deletion of WNT16 in osteoblasts results in a decrease in bone mass and quality with increased susceptibility to fractures [91]. On the other hand, osteocytes secrete factors that regulate bone mass and mineral homeostasis, including RANKL, OPG, Dickkopf-1 (DKK1), sclerostin (SOST) and fibroblast growth factor-23 (FGF23) [75]. SOST is highly expressed in osteocytes and functions as an important regulator of bone remodeling by inhibiting osteoblast differentiation and activity through antagonizing the canonical Wnt pathway and stimulating osteoclastogenesis in a RANKL-dependent manner. SOST binds to the extracellular domain of LRP5/6 that is expressed on the surface of osteoblasts and consequently inhibits the activation of the Wnt-signaling pathway which is needed for bone formation. Moreover, SOST promotes the differentiation and activity of osteoclasts through upregulating the expression of carbonic anhydrase 2 (CA2), CTSK, and ACP5. The importance of SOST as a key regulator of bone remodeling is evident in sclerosteosis, a human genetic bone disorder characterized by high bone mass due to the lack of SOST [92,93,94,95,96]. DKK1 is another LRP5/6 antagonist and exerts the same effects of SOST in vivo [97]. FGF23 is a bone-derived hormone that is produced by both osteoblasts and osteocytes and plays an essential role in bone mineralization through suppressing the secretion of PTH from the parathyroid glands, the reabsorption of phosphate and the production vitamin D (1,25(OH)2D3) in the kidney [98]. 

There is a growing body of evidence to suggest that inflammation influences maturation, as well as the growth of osteocytes and osteoblasts and is highly correlated with cell apoptosis. It should also be noted that in inflammatory conditions, both osteocytes and osteoblasts may amplify local inflammation via their ability to produce a number of pro-inflammatory cytokines and chemokines that disrupt homeostatic status in the bone microenvironment, resulting in an increase in bone resorption and a decrease in bone formation [26,99,100]. In addition to their ability to act as immune cells by producing pro-inflammatory mediators such as TNF-α, IL-6 and IL-1β, apoptotic osteocytes and osteoblasts putatively release alarmins, including the high mobility group box 1 (HMGB1) protein that also promotes inflammation and osteoclastogenesis [101,102]. 

### 2.3. Potential Contribution of Osteal Macrophages to Bone Remodeling 

**Figure 3:**
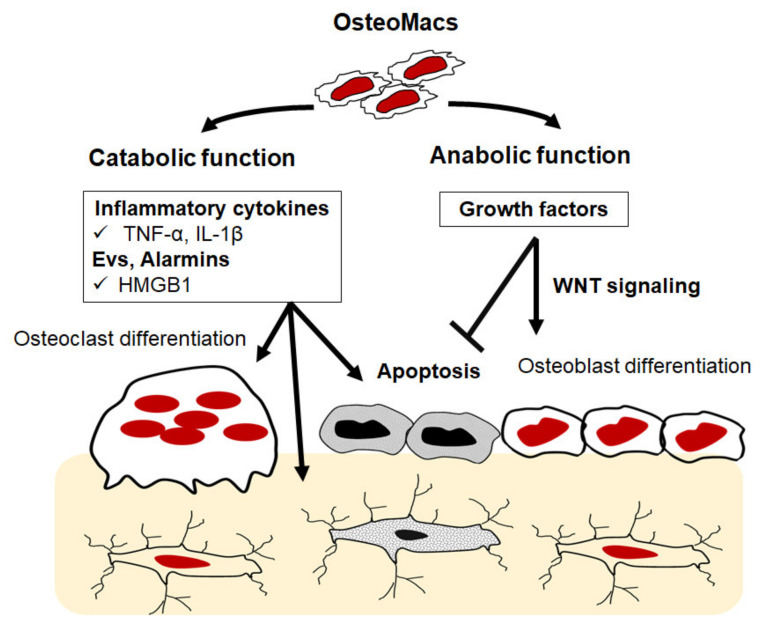
Schematic illustration of potential role of osteomacs in bone remodeling. Osteomacs can potentially produce inflammatory factors that promote osteoclastogenesis and apoptosis of osteoblasts and osteocytes. They are also able to produce anti-inflammatory and growth factors that positively regulate the function of osteoblasts and osteocytes.

Osteomacs are a special subtype of macrophage that reside in bony tissues and account for about one-sixth of the total cells in bone tissue. They are typically found in close proximity to osteoblasts and osteoclasts on the bone surface, morphologically differ from osteoclasts, and express unique cell markers including F4/80, CD68 and Mac3. Functionally, they promote osteoblast differentiation and mineralization in vitro and have been reported to enhance bone healing in fracture models in vivo [103,104,105]. The importance of osteomacs in the bone modelling process is strongly supported by the findings that macrophage depletion (achieved using macrophage Fas-induced apoptosis (Mafia)) and lysozyme M-deficient mice results in a reduction in the formation of endocortical bone and is accompanied by progressive bone loss [104,105]. Recent findings highlight the fact that osteomacs support osteoclast function through the clearance of bone resorption byproducts such as bone particulate matter and TRAP [106]. These collective findings suggest that osteomacs play an essential role in bone anabolism and in physiological bone remodeling [106]. Despite these findings, research on osteomacs is a relatively new field and many important questions remain to be answered. Generally, macrophages are a highly heterogenous population of multifunctional cells that essentially regulate tissue homeostasis and are involved in various physiological conditions and pathophysiological processes. Exposure of resident and recruited macrophages to any environmental stimuli leads to the activation of transcriptional programs that drive their differentiation into different phenotypes including pro-inflammatory M1-like and anti-inflammatory M2-like macrophages and bone-resorbing osteoclasts. Apart from being the main precursors of osteoclasts in bone tissues, macrophages, namely M1-like cells, produce an array of pro-inflammatory cytokines including TNF-α, IL-1β, IL-6, and nitric oxide (NO) that promote catabolic process in bone, as typified by the elevated osteoclastogenesis and apoptosis of osteoblasts and osteocytes [9,10,12,107,108,109,110]. In addition to factors that are secreted by macrophages, they also produce EVs that facilitate cell–cell communication within the bone microenvironment [107,110]. There is a growing body of evidence to suggest that macrophage-derived extracellular vesicles (EVs) contain various alarmins, endogenous molecules that are released upon cellular stress, that can promote local inflammation and bone loss. Among macrophage alarmins, annexin 2 (Anx2), heat shock protein 60 (HSP60), S100A and high mobility group box protein 1 (HMGB1) have been documented to potentiate the RANKL signaling pathway, resulting in increased osteoclastogenesis [109,110,111,112,113,114]. Moreover, it has also been suggested that macrophage-derived EVs contain MicroRNAs (miRNAs), small noncoding RNA molecules containing around 22 nucleotides, which function in regulating the differentiation and activity of osteoblasts and osteoclasts (Figure 3). Of these factors, miR-223 inhibits osteoblast differentiation through suppressing the expression of RUNX2 [115,116]. These collective findings suggest that an understanding of the precise function of osteal macrophages might open a new therapeutic window for bone osteolytic diseases.

## 3. Inflammation and Bone Osteolytic Diseases 

There is an increasing awareness that chronic inflammatory conditions favor a catabolic state that promotes bone loss and reduces both bone formation and bone mineral density. Bone tissues are sensitive to inflammation, and increases in the levels of pro-inflammatory cytokines such as IL-1β, IL-6 and TNF-α disrupt normal bone homeostasis via various mechanisms. Conditions with inflammatory-mediated pathological bone resorption including osteoporosis, rheumatoid arthritis, aseptic prosthesis loosening, and osteomyelitis exemplify the major skeletal disorders responsible for causing disabilities and represent significant economic burdens worldwide [4,5,6]. 

### 3.1. Osteoporosis 

Osteoporosis represents a major cause of fractures in the elderly worldwide. It is estimated that 40% of postmenopausal women and 30% of older men (over 70 years of age) are at risk of experiencing osteoporotic fractures. Menopause, aging, inflammation, and hyperparathyroidism are the leading causes of osteoporosis. The disease is characterized by low bone mass and the deterioration of bone tissue. There are no clinical symptoms until the occurrence of fractures that can be, in some cases, life-threatening [117,118,119]. Osteoporosis has been divided into primary and secondary causes. Primary osteoporosis is subdivided based on age into postmenopausal osteoporosis that occurs in women shortly after menopause, and other primary osteoporosis, present in men and women over the age of 70. Secondary osteoporosis is caused by certain lifestyle behaviors, diseases, and medications as well as alcohol abuse, smoking, poor nutrition, immobilization, gastrointestinal disease, hypercalciuria, prostate cancer and hypogonadism with decreased testosterone levels. A deficit of estrogen in postmenopausal women and a deficit estrogen, testosterone and other androgens in elderly men contribute to the development of osteoporosis, since these hormones play a regulatory role in inflammation and bone remodeling. Aging is known to be accompanied by a decrease in several physiological functions and is accompanied by an increase in metabolic risk and systematic inflammation, namely pro-inflammatory cytokines and oxidative stress [120,121,122]. More importantly, cellular senescence associated with aging is a dynamic process in which cells lose their proliferative potential, undergo irreversible growth arrest in response to stressors and stimuli and promote a distinctive pro-inflammatory secretome, termed the senescence-associated secretory phenotype (SASP). There is a growing body of evidence to suggest that the accumulation of senescent cells such as myeloid cells, osteocytes and osteoblasts facilitates the development of a pro-inflammatory environment in bone tissues, leading to osteoporosis [46,123,124]. In support of this conclusion, pharmaceutical interventions targeting the accumulation of senescent cells improves bone quality in aged mice [125]. Oxidative stress is another factor involved in the pathogenesis of bone loss during aging, since it impairs the bone remodeling process via inducing osteocyte and osteoblast apoptosis and promoting osteoclastogenesis. There is a link between the decreased levels of plasma antioxidants and bone loss found in aged or osteoporotic patients [126,127]. The current pharmacotherapies include the administration of bisphosphonates, PTH analogues, sex-hormone replacement, selective estrogen receptor modulators, RANKL inhibitors, and sclerostin inhibitors. In addition, modifying lifestyle with the objective of minimizing the risk factors associated with inflammation is considered to be key factor in the management of osteoporosis, which include a healthy balanced diet with supplementation of vitamin D, antioxidants and probiotics, regular exercise and refraining from smoking and drinking alcohol [119,127,128].

### 3.2. Rheumatoid Arthritis

Rheumatoid arthritis (RA) is one of the most widespread inflammatory arthropathies and it affects approximately 0.5–1% of the population worldwide. RA, a chronic autoimmune-mediated systemic inflammatory disease, is characterized by inflammation of the synovial joints, cartilage erosion and bone loss accompanied by pain, swelling, tenderness, stiffness, and joint deformity [129]. The immunological events leading to this type of joint damage include synovial hyperplasia with the infiltration of and the activation of lymphocytes, macrophages, and fibroblasts in the synovium, as well as the formation of aggregates of inflammatory macrophages and lymphocytes in the marrow space of the bone, resulting in synovial inflammation, which is accompanied by local bone loss (osteopenia) [129]. Of importance is the fact that an inflamed RA synovium is the major source of a number of pro-inflammatory mediators that promote the recruitment and differentiation of osteoclasts in the bone microenvironment. These include TNF-α, IL-1β, M-CSF, IL-6, IL-11, IL-17 and the parathyroid hormone-related peptide, which function to mediate osteoclast differentiation in the canonical- or non-canonical-dependent pathways [130]. Increasing the awareness that systemic inflammation in RA has an indirect, negative effect on bone mineralization through reducing the absorption of vitamin D, calcium and other minerals that are needed to maintain healthy bones [110]. Therefore, the current therapeutic interventions for RA include suppression of the primary inflammatory process in the synovial tissue. Among these strategies, TNF blockers have been shown to be effective in arresting progressive bone loss in RA patients. Monoclonal antibodies targeting IL-6 and the CD20 B-cell population have also demonstrated good effects, as evidenced by a decrease in bone degradation in RA patients [131,132,133,134]. However, lifestyle modifications should also be considered as the basis of a treatment designed to minimize risk factors associated with chronic inflammation. These changes include a low-fat low-sodium diet and supplementation with omega-3 fatty acids and probiotics. Antioxidant supplements, namely green tea, polyphenols, blueberry extracts, milk thistle extract (silymarin) and stinging nettle extract have also proven to show beneficial effects in suppressing inflammation and pain in the joints [135,136,137]. More importantly, appropriate physical activity, adequate sleep, hygiene and smoking cessation will also have positive effects on the treatment and suppression of disease progression [138]. 

### 3.3. Aseptic Loosening

Aseptic loosening due to periprosthetic inflammatory osteolysis is the most common cause of arthroplasty failure. Persistent local inflammatory response initiated by wear particles that are released from the sliding surfaces of prosthetic materials stimulates bone resorbing-osteoclast activities and bone loss around the implant, resulting in the loss of prosthesis fixation and arthroplasty failure. Macrophages recognize wear particles derived from the implant and consequently trigger a classical foreign body response, which is characterized by the release of an array of inflammatory mediators that facilitate the recruitment of inflammatory cells. Macrophages and T cells in pseudo-synovial tissue surrounding prosthetic materials represent the main source of pro-inflammatory and resorptive cytokines such as TNF-α, IL-1β, TYMP, and RANKL [139,140]. Unfortunately, neither anti-inflammatory nor anti-resorptive agents such as TNF-α and RANKL inhibitors have been shown to prevent the progression of osteolysis or prolong the lifespan of an implant, and revision surgery is the only choice for this situation. However, further exploring the inflammation and immune pathways would be expected to provide essential clues for the discovery of novel approaches for therapeutic intervention. 

### 3.4. Osteoarthritis

Osteoarthritis (OA) is a degenerative joint disease and represents a major cause of joint pain and disability in people aged ≥60 year worldwide. The prevalence of OA is expected to increase in parallel with the increase in the number of people aged ≥60 year in the world. The disease is characterized by the progressive deterioration of the articular cartilage, subchondral bone remodeling, synovial inflammation (synovitis), and hypertrophy of the joint capsule. Local inflammation, an important feature of OA, is typified by the overproduction of proinflammatory cytokines such as IL-1β, IL-6, TNF-α that might lead to the development of osteoclastogenesis and bone erosion. In early-stage OA, bone remodeling is increased, resulting in a thinner subchondral bone (bone beneath the cartilage) [141]. The decrease in bone mineral density and impaired bone microstructure including reduced trabecular thickness with increased trabecular separation in the femur and tibia result in an increase in the overload of the cartilage, leading to progressive cartilage degeneration. An elegant explanation of the pathological changes is that senescent or apoptotic osteocytes in the subchondral bone that are associated with aging promote inflammation and the RANKL pathway and inhibit bone formation and mineralization leading to thinner subchondral bone. Therefore, subchondral bone remodeling represents a potentially promising therapeutic target for osteoarthritis [141,142,143,144,145]. 

### 3.5. Osteomyelitis 

Osteomyelitis is a type of bone inflammation that is induced by infection, largely caused by the infectious organism of *Staphylococcus aureus*. Young children and the elderly with diabetes are most at risk for developing osteomyelitis. The infection may reach the bone via the bloodstream or via a local injury and consequently induces a severe inflammatory response followed by progressive bone destruction. Infection causes osteoblasts apoptosis and promotes osteoclast activities that are accompanied by an increase in RANKL expression in tissues surrounding the infection sites. Intravenous or oral antibiotic treatment for osteomyelitis is essential, and this treatment may last for many weeks. Surgical intervention is sometimes needed to drain infectious fluid and remove damaged tissues [146].

## 4. Other Mechanisms Involved in Inflammation-Induced Pathological Bone Resorption 

In addition to the direct effects of inflammation on bone remodeling, inflammation can also impact bone through different mechanisms, since it may reduce tolerance to mobility/exercise, the production of reproductive hormones, and the absorption of nutrients, calories and minerals in gastrointestinal tracts, and is the leading cause of immunosenescence and a chronic low-grade inflammatory state, all of which have been associated with bone fragility and low mineral density [8]. Physical activity is essential for bone health, owing to the fact that a balanced bone remodeling process and mineral deposition are promoted by mechanical loading. Exercise promotes muscle secretory functions that protect osteocytes from apoptosis and ameliorate the development of SASP and inflammation in the bone microenvironment [147]. On the other hand, low levels of physical activity are associated with a decline in fatty acid metabolism in adipose tissues and the secretion of adipokines accompanied by the development of systemic inflammation and insulin resistance [148]. Inflammation affects the production of leptins, a cytokine-like hormone that is secreted by adipocytes, that has anabolic functions on bone by mediating the differentiation of bone marrow stem cells into the osteoblastic cell lineage and inhibiting the differentiation of osteoclasts [149,150]. 

**Figure 4:**
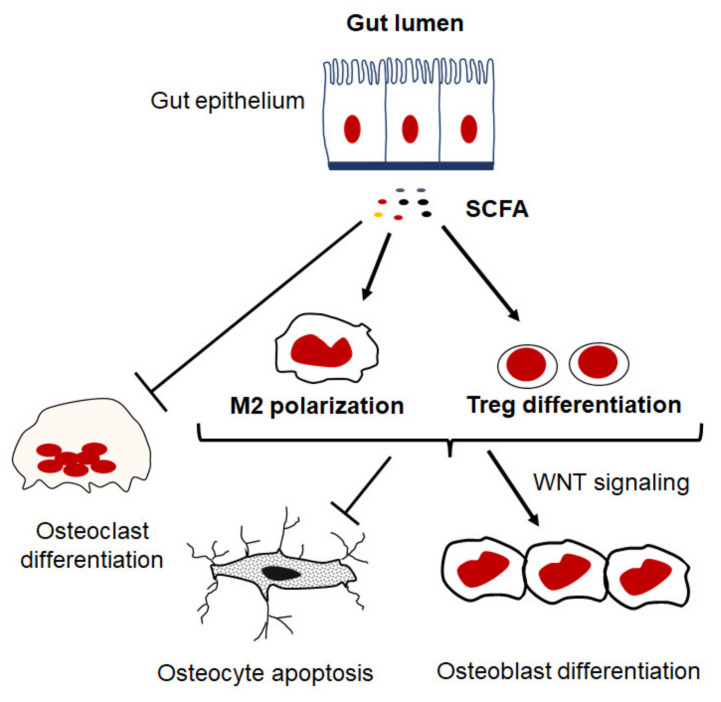
Potential regulatory role of SCFAs in inflammation and bone remodeling. SCFA, such as butyrate and propionate, inhibit osteoclastogenesis and promote differentiation of CD4 T cell into regulatory T cells (Tregs) and macrophages into an anti-inflammatory phenotype that facilitate an anabolic state in the bone microenvironment.

In certain inflammatory conditions, the intestinal barrier becomes compromised, which leads to increases in intestinal permeability and the perturbation of gut microbiota allowing foreign particles and microbiota products to enter circulation, thereby exacerbating inflammation. These processes may disrupt nutrient absorption, namely the intake of calcium, phosphate, and vitamin D, resulting in an inadequate supply for matrix formation and bone mineralization, leading to reduced bone strength. More importantly, recent findings suggest that the gut microbiota influences host inflammatory responses, gut barrier function, and nutrition intake as well as bone homeostasis [151]. Robust evidence demonstrates that alterations in microbiota composition and metabolites contribute to the development of inflammation and pathological bone loss, and rebalancing the microbiota composition by nutritional supplements with prebiotics and probiotics prevents bone loss [152]. Indeed, there is a significant correlation between the elevated levels of phylum Gemmatimonadetes and Chloroflexi, genera Blautia, Parabacteroides, Ruminococcaceae, genus Bacteroides and family Rikenellaceae in the gut microbiota and reduced bone density in osteoporosis patients compared to healthy individuals [153,154,155,156]. Metabolites produced by gut microbiota are directly involved in regulating bone remodeling either by promoting or suppressing bone-resorbing activities [152,157]. The bacterial cell wall, including peptidoglycans and lipopolysaccharides, promotes bone resorption or stimulates intestinal Th17 to produce S1P receptor 1–mediated (S1PR1-mediated), thus mediating postmenopausal bone loss [158,159]. On the other hand, hydrogen sulfide (H2S) produced by microbiota promotes bone formation via activating Wnt signaling and prevents the loss of trabecular bone in ovariectomized mice [160]. Likewise, short-chain fatty acids (SCFAs) generated by the fermentation of complex carbohydrates have recently been documented as metabolites of gut microbiota that play a key regulatory role in the skeletal system (Figure 4). Butyrate and propionate are the most important SCFAs that are known to facilitate the differentiation of CD4 T cell into regulatory T cells (Tregs) and macrophages into an anti-inflammatory phenotype resulting in an anabolic state needed for bone formation [157,161]. Consistent with the capacity of propionate and butyrate to induce bone formation, they exhibit anti-osteoclastic properties in vivo, since their treatment reduces the number of osteoclasts and prevents ovariectomy-induced bone loss [162]. Therefore, SCFAs by supplementation with probiotics have emerged as a novel therapy for preventing pathological bone loss. There is increasing evidence that demonstrates the positive effects of probiotics on bone health by increasing bone mineralization and strength in several experimental bone loss models [163,164,165,166]. Likewise, supplementation of probiotics for a period of 6-12 months has been reported to prevent pathological bone loss in clinical treatment trials for postmenopausal osteoporosis [167,168,169].

Recent findings have revealed the existence of a link between certain lifestyle factors and the development of low-grade chronic inflammation that increases susceptibility to bone diseases. Indeed, the shifts in the inflammatory response from short- to long-lived interrupts tissue homeostasis and balanced cellular physiology in organs, which lead to numerous metabolic disorders. The major lifestyle and environmental factors include diet, physical activity, psychological stress, smoking and exposure to pollutants [170]. Owing to these facts, preventive and therapeutic strategies for bone diseases should include lifestyle modifications to minimize systemic chronic inflammation. A reduction in low-grade inflammation is highly likely to be a healthy and desirable strategy and is particularly relevant to healthy aging and to improving well-being to prevent inflammageing, a process of dysregulation of innate immunity that represents a significant risk factor for morbidity and mortality in the elderly [171]. 

It is evident that diet composition contributes to the development of an inflammatory state as the consumption of carbohydrates and fats promotes inflammation, contrary to eating vegetarian diets and a Mediterranean diet, which are associated with a reduction in circulating inflammatory molecules [172,173]. This is likely attributable to the increase in pathogenic gut microbiota associated with certain diets which directly influences immune cells and inflammatory response. Therefore, a nutritional strategy with the goal of reducing inflammation should include a healthy diet with supplementation of prebiotics and probiotics. Dietary supplementation with potent anti-inflammatory supplements including omega-3 fatty acids, green tea, polyphenol, blueberry extracts, milk thistle extracts (silymarin) and stinging nettle extracts is a particularly attractive approach to combating low-grade inflammation. Such nutritional components have proven to selectively alleviate extrinsic cellular stressors, cellular senescence and consequently the inflammation state [137,174,175]. On the other hand, regular exercise and physical activity significantly contribute to the suppression of inflammation via preventing cellular senescence. Moreover, physical activity stimulates skeletal muscle and adipose tissues to produce endocrine factors that can systemically reduce inflammation [176]. Taken together, dietary and lifestyle modifications should be considered as the basis of any pharmacological intervention for bone diseases, which are characterized by excessive bone loss such as osteoporosis and RA [177]. 

## 5. Conclusions and Future Prospects

Osteolytic bone diseases constitute one of the major causes of disability and morbidity and represent an enormous economic burden for health and social care services worldwide. The pathological mechanism responsible for bone loss is complex and is affected by a variety of endocrine networks and immunological signals. To date, the majority of pharmaceutical interventions target excessive bone resorption or inflammatory cytokines. Further research directed at understanding the precise function of osteomacs in the bone remodeling process would open new windows for therapeutic intervention for bone osteolytic diseases. Moreover, it is important to uncover the molecular mechanisms that are involved in osteocyte senescence to develop a novel approach for preventing this process in bone cells for better therapeutic outcomes. Strategies to favorably diminish inflammation such as nutritional supplementation with prebiotics and probiotics and lifestyle modification programs can have a positive influence on bone health, especially in an aging population.
